# MicroRNA-181a Suppresses Mouse Granulosa Cell Proliferation by Targeting Activin Receptor IIA

**DOI:** 10.1371/journal.pone.0059667

**Published:** 2013-03-20

**Authors:** Qun Zhang, Haixiang Sun, Yue Jiang, Lijun Ding, Shaogen Wu, Ting Fang, Guijun Yan, Yali Hu

**Affiliations:** Reproductive Medicine Center, Department of Obstetrics and Gynecology, Nanjing Drum Tower Hospital, Nanjing University Medical School, Nanjing, China; Imperial College London, United Kingdom

## Abstract

Activin, a member of the transforming growth factor-β superfamily, promotes the growth of preantral follicles and the proliferation of granulosa cells. However, little is known about the role of microRNAs in activin-mediated granulosa cell proliferation. Here, we reported a dose- and time-dependent suppression of microRNA-181a (miR-181a) expression by activin A in mouse granulosa cells (mGC). Overexpression of miR-181a in mGC suppressed activin receptor IIA (acvr2a) expression by binding to its 3′-untranslated region (3′-UTR), resulting in down-regulation of cyclin D2 and proliferating cell nuclear antigen expression, leading to inhibition of the cellular proliferation, while overexpression of acvr2a attenuated the suppressive effect of miR-181a on mGC proliferation. Consistent with the inhibition of acvr2a expression, miR-181a prevented the phosphorylation of the activin intracellular signal transducer, mothers against decapentaplegic homolog 2 (Smad2), leading to the inactivation of activin signaling pathway. Interestingly, we found that miR-181a expression decreased in ovaries of mice at age of 8, 12, and 21 days, as compared with that in ovaries of 3-day old mice, and its level was reduced in preantral and antral follicles of mice compared with that in primary ones. Moreover, the level of miR-181a in the blood of patients with premature ovarian failure was significantly increased compared with that in normal females. This study identifies an interplay between miR-181a and acvr2a, and reveals an important role of miR-181a in regulating granulosa cell proliferation and ovarian follicle development.

## Introduction

It is generally accepted that follicles are the most important components of the ovary. Each follicle comprises an oocyte in the center and one or more layers of somatic granulosa cells surrounding it. Based on the size and morphology, follicles can be classified into different types, including primordial, primary, secondary, and tertiary follicles. In the primordial follicles, there is only one flat layer of granulosa cells. After recruitment of primordial follicles into the pool of growing follicles, the proliferation of granulosa cells is initiated, and the follicles begin to grow [1]–[3]. The proliferation and differentiation of granulosa cells are critical events during the development of the follicles. In addition, pituitary gonadotropins, including follicle stimulating hormone (FSH) and luteinizing hormone, are vital for the growth of the follicles and the maturation of oocytes [4], [5]. Moreover, autocrine and paracrine factors, such as transforming growth factor β1 (TGF-β1), bone morphogenetic proteins, growth and differentiation factor-9, inhibins, and activins, are secreted by oocytes or somatic cells and are important for folliculogenesis [6]–[8].

Activins, mainly produced by granulosa cells in the ovary, are indispensable for the development of ovarian follicles and for reproductive functions, as mice with genetic deletions of activin components are infertile [9]. Activins consist of two subunits (βA and βB) and have three types: activin A (βAβA), activin B (βBβB), and activin AB (βAβB). Activins are considered as feedback regulators of pituitary gonadotropin release in the ovary and positive regulators of FSH generation and secretion [10], [11]. They also regulate follicle development by promoting the growth of follicles and the proliferation of granulosa cells [12]–[14]. Like other TGF-β superfamily members, activins transduce their signal through binding to transmembrane type II receptors, activin receptor type IIA and IIB (ACVR2A and 2B). Either of ACVR2A or 2B has serine/threonine kinases activity. They may transphosphorylate the type I receptors, which in turn activate the two intracellular R-Smad signal transducers, Smad2 and Smad3. The activated R-Smads form heterodimeric complexes with Smad4, and translocate into the nucleus, where they regulate the transcription of target genes [15].

MicroRNAs (miRNAs) are 19–25 nucleotides (nt), single stranded, non-coding RNAs that bind to target mRNAs and mediate translational repression and/or mRNA degradation [16], [17]. MiRNAs control many vital biological processes, including cell proliferation and differentiation. Homozygous *Dicer-1*-deficient mice have global defects in miRNA synthesis and die during early embryogenesis [18]–[20]. However, mice with partial or conditional *Dicer-1* deletions survive to adulthood; they have been instrumental in defining specific effects of post-natal miRNA deficiency, such as those involved in female fertility and folliculogenesis [21]–[23]. Aberrant miRNA expression is associated with human diseases, including benign gynecological conditions and fertility disorders of the female reproductive tract [24], [25]. MiR-181a (5′-AACAUUCAACGCUGUCGGUGAGU-3′) is a key modulator of cellular differentiation, including hematopoietic lineage differentiation [26], myoblast differentiation [27], and T-cell sensitivity and selection [28]. Recently, Sirotkin et al. reported that miR-181a reduced proliferating cell nuclear antigen (PCNA) expression in human granulosa cells [29]. In the present study, we demonstrated that miR-181a suppressed mouse granulosa cell (mGC) proliferation by targeting activin receptor IIA (acvr2a), while overexpression of acvr2a blocked miR-181a’s inhibitory effect on mGC proliferation, indicating that miR-181a may play an important role in ovarian follicle development.

## Results

### Effect of Activin A on miR-181a Expression in mGC and on mGC Proliferation

**Figure 1:**
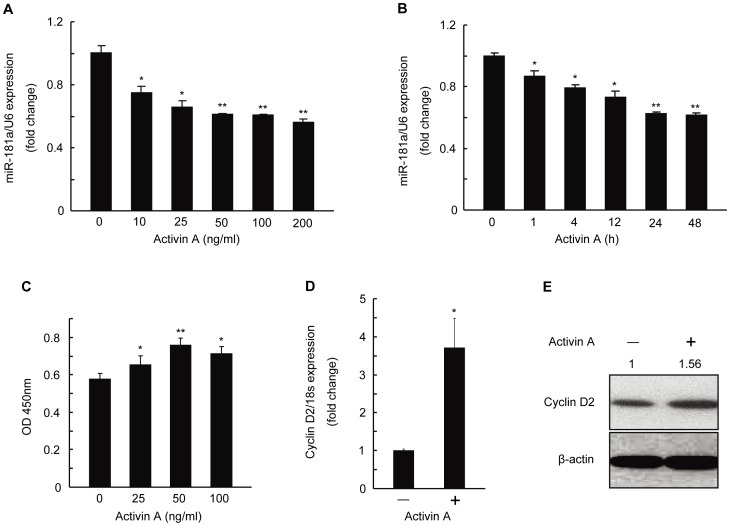
Effect of activin A on miR-181a expression and mouse granulosa cell (mGC) proliferation. mGC was isolated from 21-day-old mouse ovaries. (A) mGC was treated with indicated concentrations of activin A for 24 h. MiR-181a level was determined by qRT-PCR. (B) qRT-PCR analysis was performed to measure miR-181a level in mGC treated with activin A (50 ng/ml) for up to 48 h. (C) The proliferation of mGC was measured by CCK-8 after treated with activin A for 48 h. Cyclin D2 mRNA (D) and protein (E) levels were examined in mGC after treated with activin A (50 ng/ml) for 48 h by qRT-PCR and Western blotting, respectively. Relative protein levels were measured by densitometry using Quantity One Software and normalized to β-actin, the control group; the ratios were presented above the Western blot bands. All experiments were performed three times. *p<0.05, **p<0.01, compared with untreated controls.

Previous studies have investigated the relationship between TGF-β superfamily members and miRNAs, such as the relationship between TGF-β and miR-181 in breast cancer cells [30]. Here, to investigate the role of activin A in regulating miR-181a expression in mGC, we examined miR-181a expression in mGC after activin A treatment at various concentrations (10, 25, 50, 100, and 200 ng/ml) for 24 h. As shown in Fig. 1A, in the presence of activin A, the ratio of miR-181a to U6 was reduced; as the concentration of activin A was increasing, the reduction was more remarkable. Since we found that the higher concentration of activin A had the increasing suppression activity, but activin A at 50 ng/ml or above showed the comparable suppression (Fig. 1A), we chose 50 ng/ml activin A to treat mGC for different periods of time (1, 4, 12, 24, and 48 h, respectively). Similarly, the ratio of miR-181a to U6 in mGC was decreased more significantly when mGC was cultured for longer time (Fig. 1B). Thus activin A inhibited miR-181a expression in mGC in a dose- and time-dependent manner.

Using a commercial cell counting kit-8 (CCK-8), in which cell number can be calculated as the optical density at 450 nm (OD450) of reduced WST-8 by dehydrogenases of living cells [31], [32], we showed that activin A increased OD450 readings in the mGC culture medium (Fig. 1C), indicating that mGC treated by activin A underwent accelerated proliferation. In addition, the mRNA and protein levels of cyclin D2, which promotes granulosa cell proliferation by facilitating their transition from the G1 to S phase [33], were also increased in activin A-treated mGC (Fig. 1D and 1E).

### Inhibition of mGC Proliferation and Relevant Gene Expression in mGC by miR-181a

**Figure 2:**
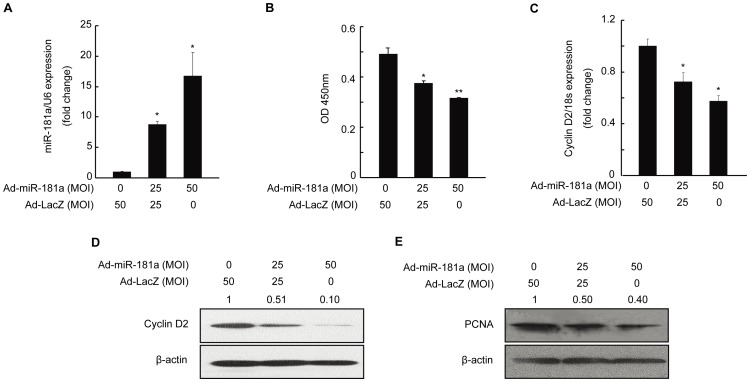
Inhibition of proliferation of and relevant gene expression in mouse granulosa cells (mGC) by miR-181a. mGC was infected with Ad-miR-181a (multiplicity of infection, MOI = 0, 25, and 50) for 48 h. (A) MiR-181a level was measured by qRT-PCR. (B) Result of a CCK-8 assay examining the proliferation of mGC. (C) qRT-PCR and (D) Western blot analysis of cyclin D2 mRNA and protein levels, respectively, in mGC. (E) Protein level of proliferating cell nuclear antigen (PCNA) as determined by Western blotting. Relative protein levels were measured by densitometry using Quantity One Software and normalized to β-actin, Ad-LacZ group; the ratios were presented above the Western blot bands. *p<0.05, **p<0.01, compared with Ad-LacZ group.

Next, we tested whether miR-181a could regulate the proliferation of mGC. After mGC was infected with adenovirus containing miR-181a (Ad-miR-181a), mature miR-181a in the cells was increased (Fig. 2A). Meanwhile, the cell number was decreased based on the reduced OD450 readings in cell count assay with the commercial CCK-8 kit (Fig. 2B).

The results of quantitative real-time PCR (qRT-PCR) analysis showed that cyclin D2 mRNA level was decreased in mGC infected with Ad-miR-181a (Fig. 2C), and Western blot results revealed that cyclin D2 and PCNA protein levels were also reduced in mGC with miR-181a overexpression in a dose-dependent manner (Fig. 2D and 2E). Moreover, we found that the proliferation of KGN cells, a human ovarian granulosa-like tumour cell line, was suppressed after overexpression of miR-181a (Fig. S1A), with a concomitant reduction of PCNA protein level (Fig. S1B). Taken together, these results demonstrate that miR-181a inhibits the proliferation of granulosa cells through suppressing the expression levels of cyclin D2 and PCNA.

### Identification of acvr2a as a Target Gene of miR-181a

**Figure 3:**
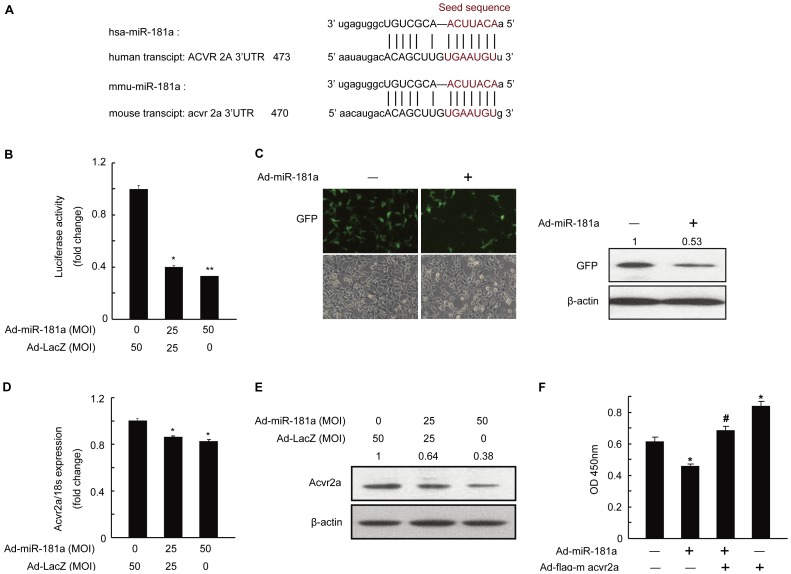
Identification of acvr2a as a target gene of miR-181a in mouse granulosa cells (mGC). (A) Putative binding sites for human (hsa) miR-181a and mouse (mmu) miR-181a in the 3′-UTR of the acvr2a gene. (B) mGC was infected Ad-miR-181a or Ad-LacZ and cotransfected with the luciferase-acvr2a-3′UTR construct. After 48 h, luciferase assays were performed. *p<0.05, **p<0.01, compared with Ad-LacZ group. (C) HEK293T cells were transfected with plasmid pEGFP-C1 carrying the 3′-UTR of acvr2a at the 3′-terminus of green fluorescent protein (GFP) alone or together with plasmid Ad-miR-181a. After 48 h, GFP expression was monitored by observation of GFP fluorescence and Western blot analysis. Acvr2a mRNA (D) and protein (E) levels in mGC were measured by qRT-PCR and Western blotting after infection of Ad-miR-181a for 48 h. *p<0.05, compared with Ad-LacZ group. (F) Result of a CCK-8 assay examining the proliferation of mGC being infected with Ad-miR-181a and/or Ad-flag-m acvr2a for 48 h. *p<0.05, compared with Ad-LacZ group; ^#^p<0.05, compared with Ad-flag-m acvr2a and Ad-miR-181a alone. Relative protein levels were measured by densitometry using Quantity One Software and normalized to β-actin, the control group; the ratios were presented above the Western blot bands.

Given that miR-181a expression was reduced in mGC treated with activin A, and miR-181a negatively influenced mGC proliferation (Fig. 2), we hypothesized that the target gene of miR-181a may be a component of the activin signaling pathway. We focused on acvr2a, one of the consistently predicted genes that contain the seed sequence of miR-181a (http://www.microrna.org) (Fig. 3A). We constructed a luciferase reporter plasmid containing the 3′-UTR of mouse acvr2a with the seed sequence (Fig. 3A) and transfected it into mGC. The transfection efficiency was approximately 54% when mGC in 60-mm dish was transfected with 3 µg pEGFP-C1 plasmid (Fig. S2). Compared to that in control group, overexpression of miR-181a in transfected mGC significantly decreased the luciferase activity (Fig. 3B), indicating that acvr2a is a target gene of miR-181a. Moreover, we subcloned the 3′-UTR of mouse acvr2a into plasmid pEGFP-C1, which contains a green fluorescent protein (GFP) transcript. Following co-transfection of HEK293T cells with this plasmid and the plasmid containing miR-181a, fluorescence microscopy and Western blotting displayed that GFP expression was decreased (Fig. 3C). Furthermore, qRT-PCR and Western blot results showed that acvr2a mRNA and protein levels were decreased in mGC infected with Ad-miR-181a (Fig. 3D and 3E). To identify whether mouse acvr2a was involved in miR-181a’s inhibitory function in mGC, we infected mGC with Ad-miR-181a and/or Ad-flag-m acvr2a. As shown in Fig. 3F, overexpression of mouse acvr2a in mGC apparently enhanced the proliferation of mGC. Particularly, increased expression of acvr2a in mGC reversed the inhibitory effect of miR-181a on mGC proliferation (Fig. 3F). Thus, all these data suggest that miR-181a inhibits mGC proliferation by targeting acvr2a.

To clarify whether the miRNA-target relationship between miR-181a and acvr2a also exists in KGN cells, we performed luciferase assay, qRT-PCR, and Western blotting, and found that miR-181a also targets ACVR2A in KGN cells (Fig. S3).

### Effect of miR-181a Inhibitor on mGC Proliferation

**Figure 4:**
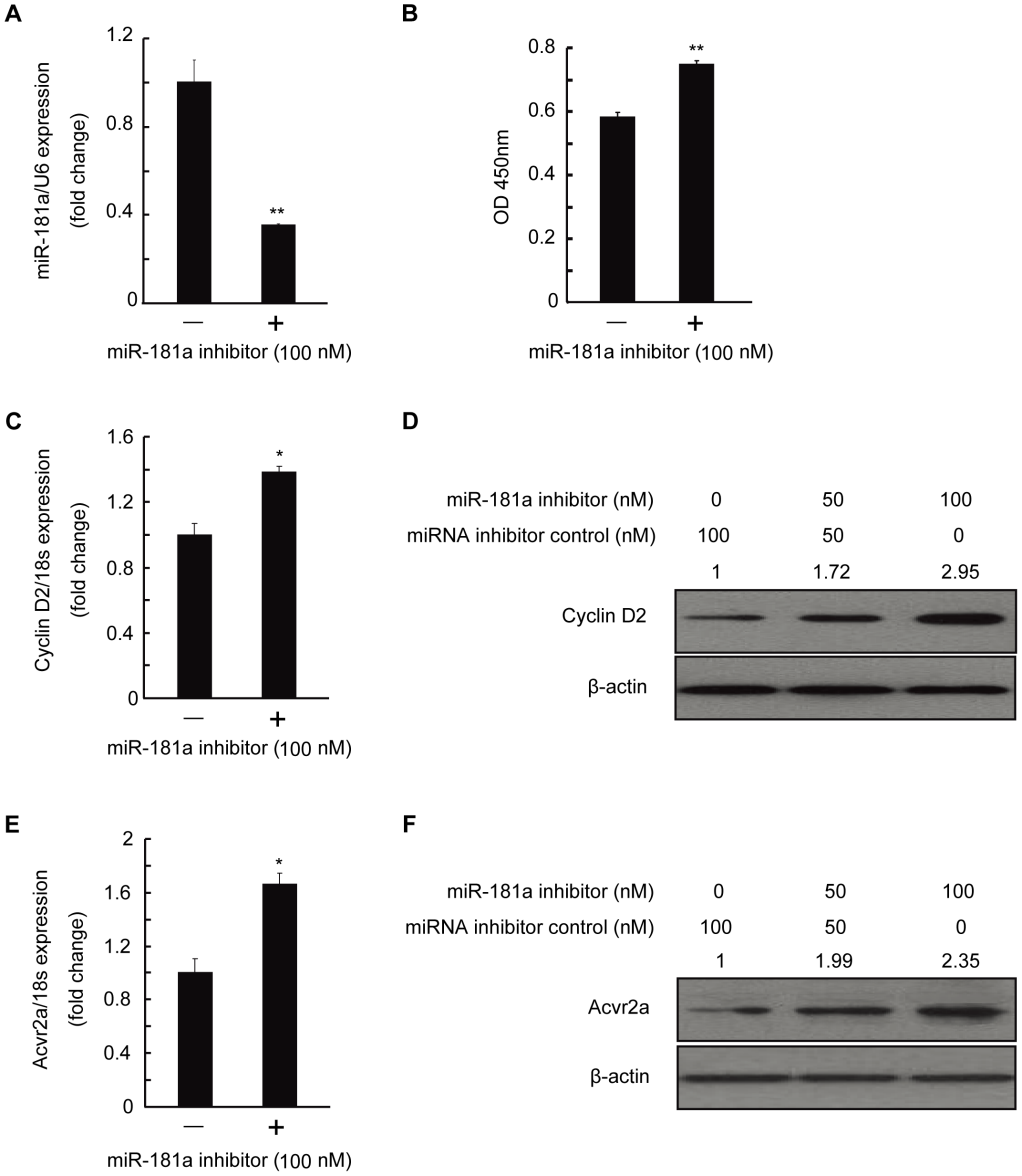
Effect of miR-181a inhibitor on mouse granulosa cell (mGC) proliferation. mGC was transfected with indicated miR-181a inhibitor (anti-sense oligonucleotide of miR-181a) or miRNA inhibitor negative control (miRNA inhibitor control) for 48 h. (A) MiR-181a expression was measured by qRT-PCR. (B) The proliferation of mGC was examined by CCK-8 after transfection of miR-181a inhibitor. Cyclin D2 mRNA (C) and protein (D) levels measured by qRT-PCR and Western blotting. (E) qRT-PCR and (F) Western blot analysis showed acvr2a mRNA and protein levels in mGC treated with miR-181a inhibitor. Relative protein levels were measured by densitometry using Quantity One Software and normalized to β-actin, the control group; the ratios were presented above the Western blot bands. *p<0.05, **p<0.01, compared with controls.

To verify above results from a different standpoint, we transfected mGC with a synthesized anti-sense oligonucleotide of miR-181a. qRT-PCR analysis revealed that miR-181a expression was reduced (Fig. 4A), CCK-8 assay showed that the proliferation of mGC was enhanced (Fig. 4B), and qRT-PCR and Western blot results displayed that cyclin D2 expression was up-regulated (Fig. 4C and 4D). Accordingly, the expression of acvr2a was also enhanced (Fig. 4E and 4F). These data support that miR-181a inhibits mGC proliferation by targeting acvr2a.

### Inactivation of the Activin Signaling Pathway by miR-181a

**Figure 5:**
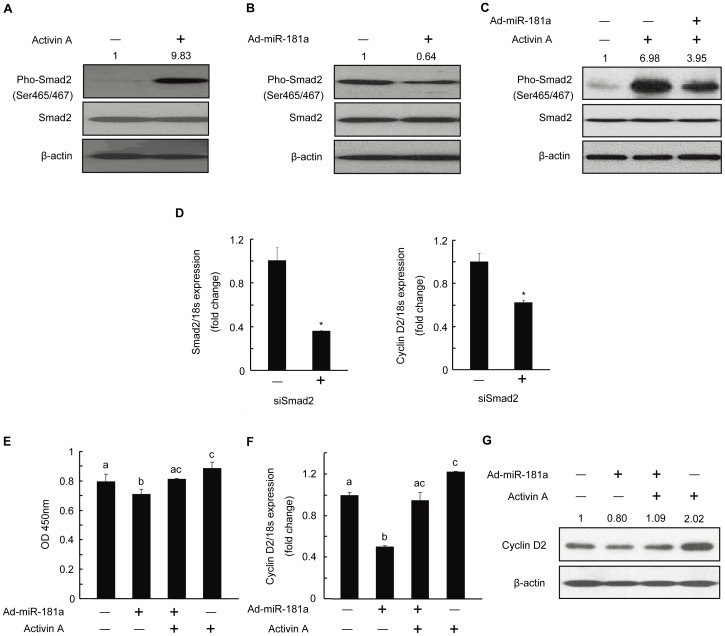
Inactivation of the activin signaling pathway by miR-181a. Western blot analysis of the levels of Smad2 and phosphorylated Smad2 (Ser465/467) in mouse granulosa cells (mGC) treated with 50 ng/ml activin A (A) or infected with 50 MOI Ad-miR-181a (B) for 24 h. (C) mGC was infected with 50 MOI Ad-miR-181a for 24 h, and cells were then treated with 50 ng/ml activin A for another 24 h. The protein level of Smad2 and phosphorylated Smad2 were measured by Western blotting. (D) qRT-PCR analysis of Smad2 and cyclin D2 expression in mGC transfected with 50 nM siRNA duplexes targeting mouse Smad2 (siSmad2) or siRNA negative control for 48 h. *p<0.05, compared with controls. (E, F, and G) mGC was infected with 50 MOI Ad-miR-181a or Ad-LacZ for 24 h, and cells were then treated with 50 ng/ml activin A for another 24 h. (E) mGC proliferation was examined by CCK-8. Cyclin D2 mRNA (F) and protein (G) levels were measured by qRT-PCR and Western blotting. Relative protein levels of phosphorylated Smad2 and cyclin D2 were measured by densitometry using Quantity One Software and normalized to β-actin, the control group; the ratios were presented above the Western blot bands. Bars labeled with different letters indicate statistically significant differences (p<0.05).

Since activins function by binding to ACVR2A or ACVR2B, and activating intracellular Smads via phosphorylation [15], we tested whether this function could be attenuated by miR-181a. We treated mGC with activin A and performed Western blotting to measure the level of Smad2 phosphorylation. As previously reported, activin A dramatically induced Smad2 phosphorylation, but the Smad2 protein level did not change (Fig. 5A). The examination of phosphorylated Smad2 level in both mGC and KGN cells infected with Ad-miR-181a revealed that miR-181a suppressed Smad2 phosphorylation in both cell types (Fig. 5B and Fig. S4). We also infected mGC with Ad-miR-181a before activin A stimulation. As shown in Fig. 5C, the effect of activin A on Smad2 phosphorylation was apparently attenuated by miR-181a, but the protein levels of Smad2 was not affected by miR-181a or activin A (Fig. 5C). After knockdown of Smad2 by transfection of mGC with a Smad2 siRNA, cyclin D2 mRNA level was reduced (Fig. 5D). Consistent with the inhibition of Smad2 phosphorylation by miR-181a, the promoting effect of activin A on mGC proliferation was inhibited by miR-181a overexpression (Fig. 5E). Furthermore, we observed that activin A-induced cyclin D2 expression in mGC was significantly suppressed by the overexpression of miR-181a (Fig. 5F and 5G).

### Regulation of Activin-induced Genes by miR-181a

**Figure 6:**
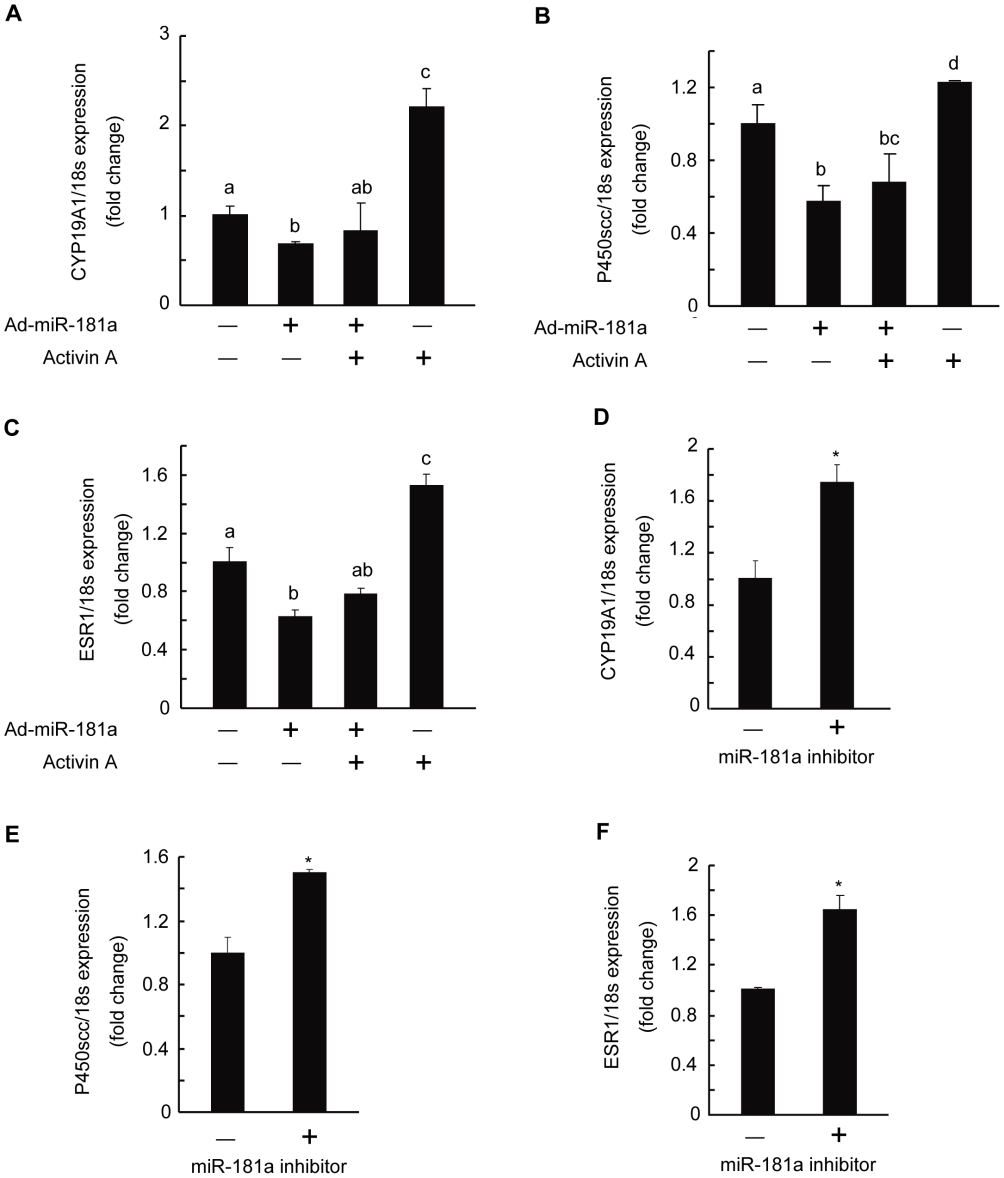
Regulation of activin-induced genes by miR-181a. (A, B, and C) Mouse granulosa cells (mGC) were infected with 50 MOI Ad-miR-181a or Ad-LacZ for 24 h, followed by 50 ng/ml activin A stimulation for another 24 h. CYP19A1, P450scc, and ESR1 levels were measured by qRT-PCR. Bars labeled with different letters indicate statistically significant differences (p<0.05). (D, E, and F) qRT-PCR analysis of CYP19A1, P450scc, and ESR1 expression in mGC transfected with 100 nM miR-181a inhibitor for 24 h. *p<0.05, compared with controls.

Previous studies have identified many genes regulated by activin A in granulosa cells, such as CYP19A1, P450scc, and ESR1 [34]–[36]. Here we investigated the suppressive effect of miR-181a on activin A-induced gene expression. Our results showed that the mRNA of CYP19A1, P450scc, and ESR1 in mGC was modestly enhanced by activin A, and simultaneous expression of miR-181a reversed this enhancement (Fig. 6A, 6B, and 6C). MiR-181a inhibitor also increased CYP19A1, P450scc, and ESR1 gene expression in mGC (Fig. 6D, 6E, and 6F).

### Variation of miR-181a and acvr2a Expression in Development of Ovaries and during Ovarian Follicle Maturation in Neonatal Mice

**Figure 7:**
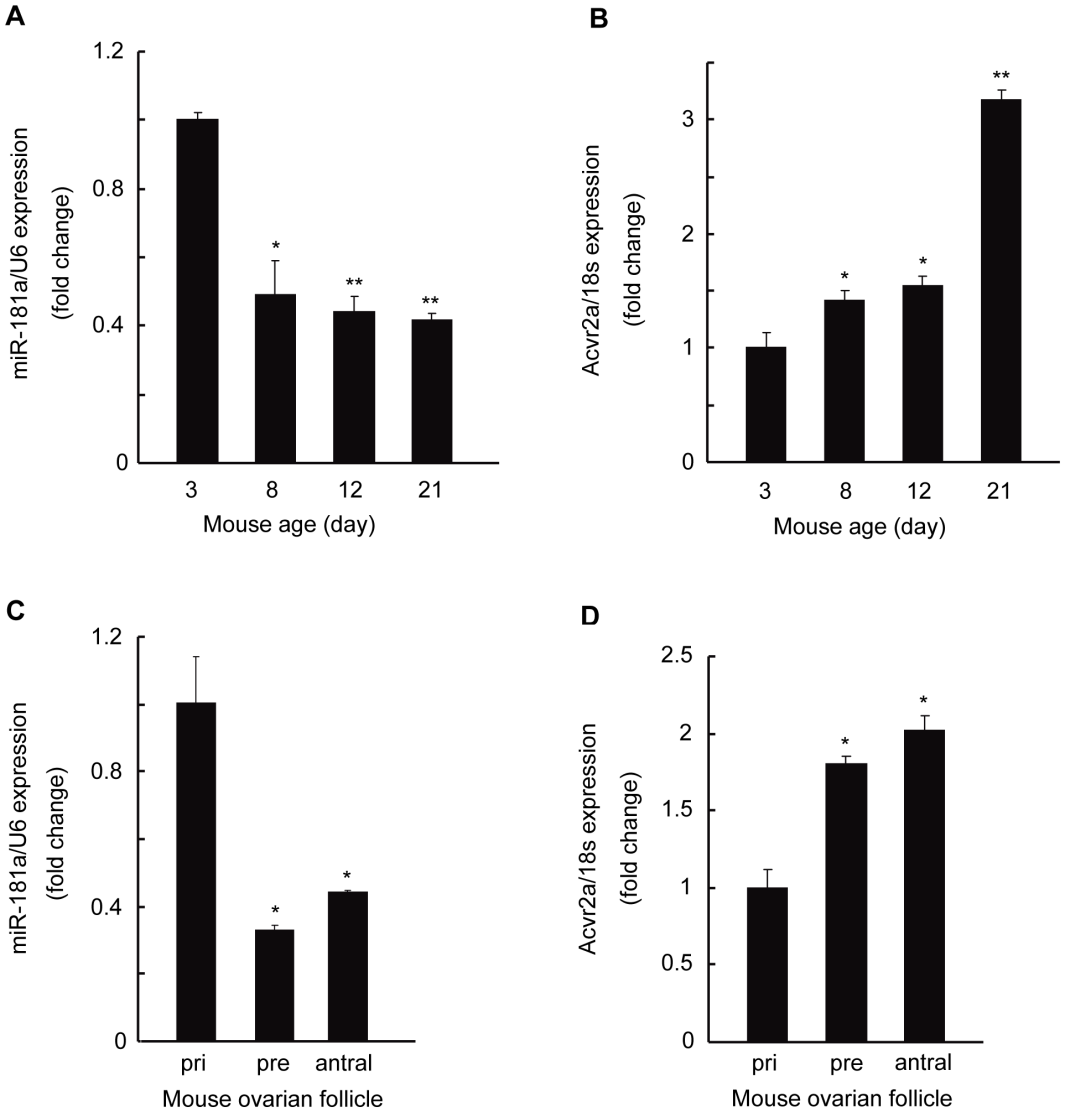
Variation of miR-181a and acvr2a expression in development of ovaries and during ovarian follicle maturation. Expression of miR-181a (A) and acvr2a (B) assessed by qRT-PCR in day 3, 8, 12, and 21 mouse ovaries. *p<0.05, **p<0.01, compared with the day 3 group. qRT-PCR analysis of miR-181a (C) and acvr2a (D) in primary (pri), preantral (pre), and antral follicles of 21-day-old mouse ovaries. *p<0.05, compared with primary follicles.

To further investigate the relationship between miR-181a and acvr2a during ovarian development, we examined their expression profiles in mouse ovaries and follicles. The miR-181a and acvr2a levels in ovaries from mice at various ages (day 3, 8, 12, and 21) were assessed. qRT-PCR analysis revealed that, compared with that at day 3, the expression of miR-181a was decreased at day 8, 12, and 21 respectively (Fig. 7A), whereas acvr2a expression was increased (Fig. 7B). We also extracted total RNA from whole follicles at various stages. qRT-PCR results showed that, compared with the expression in primary follicles, miR-181a expression in preantral and antral follicles was decreased (Fig. 7C), while acvr2a levels were increased (Fig. 7D).

### Increased miR-181a Levels in the Blood of Premature Ovarian Failure Patients

**Figure 8:**
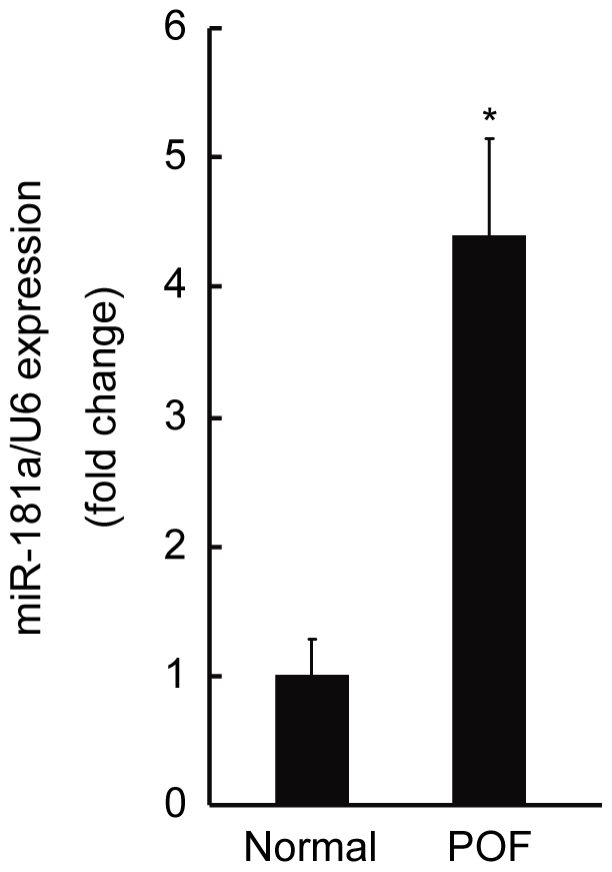
MiR-181a levels in premature ovarian failure (POF) patients and normal females. qRT-PCR analysis of miR-181a in the blood of POF patients (n = 8) and of normal females (n = 11). *p<0.05, compared with normal females.

Since the results in the present study indicate that miR-181a plays an important role in granulosa cell proliferation and ovarian follicle development, we speculated whether patients with ovarian dysfunction and reproductive diseases had abnormal expression of miR-181a. We examined miR-181a levels in the blood of normal females (n = 11) and patients (n = 8) with premature ovarian failure (POF) in whom FSH levels were elevated (Table S1). qRT-PCR analysis showed that miR-181a levels were significantly enhanced in POF patients (Fig. 8). The findings imply that miR-181a may be involved in the pathogenesis of POF.

## Discussion

Normal proliferation and differentiation of granulosa cells are critical for the development of ovarian follicles, as abnormal growth of granulosa cells will lead to infertility [37]–[39]. Despite the elucidated role of many genes such as activins in promoting the proliferation of granulosa cells and the growth of preantral follicles, little is known about their post-transcriptional regulation mechanisms. Herein, we identified that miR-181a suppresses mGC proliferation through binding to the 3′-UTR of acvr2a. This effect is supported by our findings: activin A down-regulated miR-181a expression in mGC; overexpression of miR-181a suppressed mGC proliferation; miR-181a inhibitor promoted mGC proliferation; miR-181a bound to the 3′-UTR of acvr2a resulting in reduced expression of acvr2a in mGC; overexpression of acvr2a blocked miR-181a’s inhibitory effect on mGC proliferation.

Recently, various miRNAs have been found to be involved in the control of ovarian function. Fiedler et al. reported that miR-132, miR-212, and miR-21 in mGC were up-regulated by LH/hCG [40]. MiR-21 was identified to block the apoptosis of mouse periovulatory granulosa cells [41]. More recently, miR-26b and miR-23a were found to promote granulosa cell apoptosis [42], [43]. In the present study, we demonstrated that miR-181a exerted a suppressive effect on mGC proliferation. MiR-181a has been found to be abundant and play a bifunctional role in primordial germ cells: inhibiting their somatic differentiation and preventing them from entering meiosis [44]. Lingenfelter et al. also reported that miR-181a may have an important role in oocytes by targeting nucleoplasmin 2 [45]. Our study indicates that adenovirus mediated miR-181a overexpression inhibited mGC proliferation. MiR-181a mimics, synthesized oligonucleotides, were also found to suppress mGC proliferation (unpublished data), suggesting that inhibition of mGC proliferation should be closely associated with miR-181a, rather than the toxic effect of Ad-miR-181a.

Acvr2a is necessary for reproductive development, as acvr2a-deficient mice display defective reproductive performance [46]. Acvr2a ectodomains may also suppress activin A-induced mGC proliferation by blocking the function of acvr2a [6]. Our study showed that miR-181a suppressed acvr2a expression by targeting its 3′-UTR (Fig. 3). Interestingly, the protein level of acvr2a in mGC was dramatically decreased by miR-181a, whereas the mRNA level was only slightly reduced. This indicates that through binding to the 3′-UTR of acvr2a, miR-181a has more powerful influences on acvr2a translation inhibition than mRNA degradation in mGC. The negative regulation of acvr2a expression suggests that miR-181a may play an important role in the activin signaling pathway.

The downstream signaling pathway of activins is mediated by phosphorylation of Smad2/3, which translocate into the nucleus to regulate the expressions of target genes, such as cyclin D2 [35]. Previous study showed that blocking the phosphorylation of Smad2/3 using the ALK4/5/7 inhibitor completely ablated oocyte secreted factor-induced effects on cumulus cell expansion [47], suggesting the importance of this pathway. Our results showed that miR-181a inhibited the phosphorylation of Smad2 on Ser^465/467^ (Fig. 5) and that miR-181a decreased the expression of cyclin D2 and PCNA in mGC, leading to an attenuation of cell proliferation (Fig. 2). These results further support miR-181a as a critical regulator in the proliferation of granulosa cells.

Studies have revealed that in 3-day-old mouse ovaries, many primordial follicles appear with several flat granulosa cells surrounding oocytes [2]. In the ovaries of 7-day-old mice, granulosa cells become square, leading to the formation of primary follicles. This process initiates the proliferation of granulosa cells, and many secondary follicles are formed in 12–14-day-old mouse ovaries [2], [48]. In the present study, we found that miR-181a expression was markedly reduced in 8, 12, and 21-day-old mouse ovaries, in which granulosa cell proliferation is initiated and activated, while acvr2a level was increasing (Fig. 7A and 7B). However, it is not clear why acvr2a level was apparently increased in 21-day-old mouse ovaries compared with 8 and 12-day-old ones while miR-181a had little change in them. The discrepancy may be due to that there are other kinds of cells besides mGC in the ovaries or that acvr2a expression in vivo is complicatedly regulated by many factors.

Furthermore, compared with that in primary follicles, miR-181a expression was reduced in preantral follicles, where granulosa cell proliferation was activated, whereas acvr2a expression was abundant in them (Fig. 7C and 7D). These results suggest that miR-181a may inhibit the proliferation of granulosa cells and the development of follicles *in vivo*, and we will do further study to identify this regulation effect in the future. In addition, the level of miR-181a was also lower in antral follicles, which contain differentiated, steroidogenic granulosa cells. This indicates that miR-181a plays a potential role in regulating the steroidogenesis of granulosa cells. Consistent with this hypothesis, CYP19A1, which encodes the key enzyme for estrogen biosynthesis, was found to be down-regulated by miR-181a in mGC (Fig. 6A).

POF is resulting from follicle dysfunction or depletion [49]. In most cases, an unknown mechanism leads to abnormal folliculogenesis. Recently, we found that miR-181a expression was much higher in the blood of POF patients by microarray study (unpublished data). Using qRT-PCR, we verified the increased level of miR-181a in the blood of POF patients (Fig. 8). Therefore, this study may help us to understand the causative factors that are involved in the development of POF.

In conclusion, the present study has demonstrated that miR-181a inactivates activin-induced granulosa cell proliferation by down-regulating the expression of acvr2a. The relationship between miR-181a and acvr2a in mGC proliferation helps us understand the mechanisms of follicle development at a post-transcriptional level, which may be involved in the pathology of POF.

## Materials and Methods

### Animals

Three-week-old ICR mice were purchased from the Experimental Animal Center of Yangzhou University (Yangzhou, China) and maintained in the Animal Laboratory Center of Nanjing Drum Tower Hospital (Nanjing, China) on a 12/12 h light/dark cycle (lights off at 19∶00) with food and water available ad libitum. The animal experiments were approved (SYXK 2009-0017) by the Institutional Animal Care and Use Committee at the Nanjing Drum Tower Hospital, Nanjing University Medical School.

### Cell Lines

The KGN cells, derived from human ovarian granulosa-like tumour, were cultured as previously described [50]. Human embryonic kidney cells, HEK293T and HEK293A cells, were separately maintained in DMEM supplemented with 10% FBS (HyClone, Thermo Scientific, UT, USA) and 100 IU/ml penicillin and 100 µg/ml of streptomycin, at 37°C in a humidified environment with 5% CO_2_.

### Granulosa Cell Isolation and Culture

mGC was collected from the ovaries of 21-day-old immature ICR mice using the follicular puncture method as described previously [36]. The mice were not treated with diethylstilboestrol (DES) prior to isolation, since DES may suppress the expression of activins [51]. The isolated cells, with 95% purity judged by follicle stimulating hormone receptor (FSHR) staining (Fig. S5), were greater than 95% viability as determined by Trypan blue exclusion. The cells were conventionally cultured in DMEM/F12 medium (Gibco BRL/Invitrogen, Carlsbad, CA, USA) supplemented with 10% FBS, 1 mM sodium pyruvate, 2 mM glutamine, 100 IU/ml penicillin, and 100 µg/ml streptomycin. The cells were cultured without FSH addition to avoid its effect on mGC proliferation. In some experiments, the cells were culture in the presence of recombinant human/mouse/rat activin A (Catalog Number: 338-AC, R&D Systems, Minneapolis, MN, USA). The cells were used within four passages.

### Generation of Recombinant Adenovirus

Adenoviruses harboring a 456-bp DNA fragment encompassing the hsa-miR-181a gene (Ad-miR-181a) and Flag-tagged mouse acvr2a (Ad-flag-m acvr2a) were generated using the AdMax (Microbix Biosystems, Inc., Toronto, Canada) and pSilencer™ adeno 1.0-CMV (Ambion, Austin, TX, USA) systems. The adenovirus bearing LacZ (Ad-LacZ) was obtained from Clontech (Palo Alto, CA, USA) and used as the control in the miR-181a overexpression experiments [52], [53]. The viruses were packaged and amplified in HEK293A cells and purified using CsCl banding followed by dialysis against 10 mM Tris-buffered saline with 10% glycerol. The viral titer was determined using HEK293A cells and the Adeno-X Rapid Titer kit (Clontech) [54].

### Oligonucleotide Transfection

#### MiR-181a inhibitor

(5′-mAmCmUmCmAmCmCmGmAmCmAmGmCmGmUmUmGmAmAmUmGmUmU-3′), miRNA inhibitor negative control, siRNA duplexes target mouse Smad2 (5′-GCUGAGUGCCUAAGUGAUAdTdT-3′), and siRNA negative control (5′-CGUACGCGGAAUACUUCGAdTdT-3′) were synthesized by Ribobio (Guangzhou, China). Both miRNA inhibitor negative control and siRNA negative control share no homologous region with the mouse genome sequences. Oligonucleotide transfection was performed in mGC with Lipofectamine 2000. For each transfection, 50 or 100 nM of miR-181a inhibitor or siRNA was added to each well on the six-well plate according to the protocol of the manufacturer.

### Luciferase Reporter Assay

Based on the human and mouse acvr2a mRNA sequences in GenBank (accession nos. NM_001616.3 and NM_007396.4), firefly luciferase cDNA fused with the human 3′-UTR of the ACVR2A gene (nt 284 to 733) or the mouse 3′-UTR of the acvr2a gene (nt 1 to 605) was amplified separately from the genomic DNA of human and mouse granulosa cells and cloned into the pGL3-promoter luciferase reporter vector using Xba I restriction sites. The primers for the human and mouse 3′-UTR region of ACVR2A were 5′-CTAGTCTAGATTTGGACCTGGCTAATGGAG-3′ and 5′-CTAGTCTAGAGGCCACTTATTGTTGGCACT-3′, and 5′-TATATCTAGATGGTGGCACCGTCTGTACAC-3′ and 5′-GATATCTAGATAGCAACCGTGGAACTGAGG-3′, respectively. Preconfluent (60 to 70%) mGC in six-well plates was infected with Ad-miR-181a and then transfected with 300 ng of the firefly luciferase reporter plasmid (Luc-acvr2a-3′-UTR) and 20 ng of the Renilla luciferase reporter plasmid, pRL-RSV (Promega), using Lipofectamine 2000 transfection reagent. Preconfluent (60 to 70%) HEK293T cells in six-well plates were transfected with 300 ng of the firefly luciferase reporter plasmid (Luc-ACVR2A-3′-UTR) and 20 ng of the Renilla luciferase reporter plasmid, pRL-RSV, using Nanofectin (PAA, Pasching, Austria). After 48 h, the cell lysates were assayed for luciferase activity using the Luciferase Assay System (Promega), and the activity was measured using a luminescence counter (Centro XS3 LB 960, Berthold Technologies). Firefly luciferase activity was normalized for transfection efficiency with the corresponding Renilla luciferase activity. All transfection experiments were performed at least 5 times.

### Construction of pEGFP-C1 m acvr2a 3′-UTR Plasmid

The amplified mouse 3′-UTR of the acvr2a gene (nt 1 to 605) was cloned into the untranslated 3′-terminus of GFP in the pEGFP-C1 vector. Preconfluent (60 to 70%) HEK293T cells in 60-mm dish was transfected with 1 µg of the GFP expression plasmid (pEGFP-C1 m acvr2a 3′-UTR) and Ad-miR-181a construct using Nanofectin. At 48 h post-transfection, GFP protein was detected by fluorescence microscopy and Western blotting.

### RNA Isolation and Quantitative Real-time PCR

Total RNA was extracted from cultured cells or tissues using the Trizol reagent (Invitrogen, Carlsbad, CA, USA), and the RNA integrity was assessed using denaturing formaldehyde gel electrophoresis. Total RNA (2 µg) was reverse-transcribed to cDNA using a PrimeScript RT reagent kit (BIO-RAD, Hercules, CA, USA), and qRT-PCR was performed on a MyiQ Single-Color Real-Time PCR Detection System (BIO-RAD). To detect miR-181a expression, cDNA was synthesized using the following miR-181a-specific stem-loop primer: 5′-CTCAACTGGTGTCGTGGAGTCGGCAATTCAGTTGAGACTCACCG-3′ as previously described [55]. For qRT-PCR analysis of miR-181a, we used the following primers: forward, 5′-ACACTCCAGCTGGGAACATTCAACGCTGTCG-3′; reverse, 5′-GGTGTCGTGGAGTCGGCAATTCAGTTGAG-3′. The small nuclear RNA, U6, was used as an internal control and was amplified with the following primers: forward, 5′-CTCGCTTCGGCAGCACA-3′; reverse, 5′-AACGCTTCACGAATTTGCGT-3′. The qRT-PCR conditions were as follows: 95°C for 15 min followed by 40 cycles at 95°C for 15 sec and 60°C for 1 min. The specific primers used for acvr2a, cyclin D2, CYP19A1, P450scc, ESR1, and 18s rRNA detection were listed in supplemental Table 2 (Table S2). 18s rRNA has been identified to be a more stable reference gene in ovary [56], [57]. Samples were run in duplicate with RNA preparations from three independent experiments. The fold change of each gene expression was calculated using the 2^−△△CT^ method with 18S rRNA or U6 as an internal control [58].

### Western Blotting

Protein extracts prepared from cultured granulosa cells were analyzed by Western blotting as previously described [59]. Briefly, cells were rinsed with ice-cold PBS (pH 7.4) and lysed with lysis buffer (50.0 mmol/L Tris, pH 7.6, 150.0 mmol/L NaCl, 0.1% SDS, 1.0% NP-40, and protease and phosphatase inhibitor cocktails (Sigma, St. Louis, MO, USA)). Protein concentrations were determined by Bradford assay (Bio-Rad). Equal amounts of total proteins (20–30 µg) were separated on a 10% SDS-polyacrylamide gel and transferred onto polyvinylidene fluoride membranes (Millipore, Billerica, MA, USA). Immunoblotting was performed with primary antibodies against cyclin D2 (1∶1,000; Cell Signaling Technology, Danvers, MA, USA), phospho-Smad2 (Ser465/467; 1∶1,000; Cell Signaling Technology; also detects phosphorylated Smad3 at Ser423/425), Smad2 (1∶1,000; Cell Signaling Technology), PCNA (1∶500; Santa Cruz Biotechnology, Santa Cruz, CA, USA), or acvr2a (1∶500; Santa Cruz Biotechnology). β-actin (1∶10,000; Abcam, Cambridge, MA, USA) was measured as an internal control. Immunodetection was accomplished using a goat anti-rabbit (1∶5000; Bio-Rad) or rabbit anti-mouse (1∶10,000; Bio-Rad) HRP conjugated secondary antibody and an enhanced chemiluminescence kit (Amersham Biosciences Corp., Piscataway, NJ, USA).

### Cell Proliferation Assay

Granulosa cells were seeded into 96-well plates at approximately 5,000 cells per well, cultured in growth medium (100 µl per well) and infected with adenovirus or miR-181a inhibitor. Cell proliferation assays were performed using a cell counting kit-8 assay (Dojindo Laboratories, Kumamoto, Japan). After incubation for 48 h, 10 µl of CCK-8 solution was added to each well, and the cells were incubated for another 2 h. The cell number was calculated in a 96-well format plate reader (Thermo Electron Corp, Taunton, MA, USA) done in triplicate by measuring the OD450.

### FSH Determination

All POF patients were selected from the Nanjing Drum Tower Hospital, Nanjing University Medical School, and informed consent was obtained from all participants. The FSH levels in blood were measured using chemiluminescent immunoassay (CLIA) with the sensitivity of 0.2 IU/L (Beckman Coulter, Brea, CA, USA).

### Statistical Analysis

In this study, each experiment was performed at least three times. Data are presented as the means ± SD. Statistical analysis was performed by ANOVA followed by the Student-Newman-Keuls test for experiments involving more than two groups. Student’s t-test was performed for comparison with two groups. *P* values <0.05 were considered to be statistically significant.

## Supporting Information

Figure S1 **Effect of miR-181a on KGN cell proliferation.** KGN cells were infected with Ad-miR-181a (MOI = 0, 25, and 50) for 48 h. (A) The proliferation of KGN cells was measured by CCK-8. (B) Protein level of PCNA was examined by Western blotting. Relative protein levels were measured by densitometry using Quantity One Software and normalized to β-actin, Ad-LacZ group; the ratios were presented above the Western blot bands. *p<0.05, **p<0.01, compared with control groups.
(TIF)

Figure S2 **Transfection efficiencies of mGC detected by FCM after transfection with pEGFP-C1 plasmid for 48 h.** mGC in 60-mm dish was transfected with 3 µg pEGFP-C1 plasmid. After 48 h, GFP fluorescence (A) and flow cytometry (B) were performed to measure the transfection efficiencies of mGC.
(TIF)

Figure S3 **Identification of ACVR2A as a target gene of miR-181a in KGN cells.** (A) The 3′-UTR luciferase activity of the human ACVR2A gene was examined in HEK293T cells after overexpression of miR-181a. ACVR2A mRNA (B) and protein (C) levels were measured by qRT-PCR and Western blotting in KGN cells infected with Ad-miR-181a for 48 h. Relative protein levels were measured by densitometry using Quantity One Software and normalized to β-actin, Ad-LacZ group; the ratios were presented above the Western blot bands. *p<0.05, **p<0.01, compared with controls.
(TIF)

Figure S4 **Inhibition of the phosphorylation of Smad2 in KGN cells by miR-181a.** Western blot analysis of the levels of Smad2 and phosphorylated Smad2 (Ser465/467) in KGN cells treated with Ad-miR-181a for 24 h. Relative protein levels of phosphorylated Smad2 were measured by densitometry using Quantity One Software and normalized to β-actin, Ad-LacZ group; the ratios were presented above the Western blot bands.
(TIF)

Figure S5 **Measurement of follicle stimulating hormone receptor (FSHR) by immunofluorescent staining.** Primary granulosa cells were isolated from 21-day-old mouse ovaries. FSHR protein was measured by immunofluorescent staining (FSHR: red; DAPI: blue).
(TIF)

Table S1 FSH levels in premature ovarian failure (POF) patients and normal females.
(DOC)

Table S2 Oligonucleotide primer sequences of quantitative real-time PCR.
(DOC)

## References

[1] Picton HM, Harris SE, Muruvi W, Chambers EL (2008) The in vitro growth and maturation of follicles. Reproduction 136: 703–715. 19074213 10.1530/REP-08-0290

[2] Peters H (1969) The development of the mouse ovary from birth to maturity. Acta Endocrinol (Copenh) 62: 98–116. 5394354 10.1530/acta.0.0620098

[3] Peters H, Byskov AG, Himelstein-Braw R, Faber M (1975) Follicular growth: the basic event in the mouse and human ovary. J Reprod Fertil 45: 559–566. 128630 10.1530/jrf.0.0450559

[4] Cayo-Colca IS, Yamagami Y, Phan TC, Miyano T (2011) A combination of FSH and dibutyryl cyclic AMP promote growth and acquisition of meiotic competence of oocytes from early porcine antral follicles. Theriogenology 75: 1602–1612. 21354603 10.1016/j.theriogenology.2010.12.023

[5] Zamah AM, Hsieh M, Chen J, Vigne JL, Rosen MP, et al (2010) Human oocyte maturation is dependent on LH-stimulated accumulation of the epidermal growth factor-like growth factor, amphiregulin. Hum Reprod. 25: 2569–2578. 10.1093/humrep/deq212 PMC2939758 20719813

[6] Gilchrist RB, Ritter LJ, Myllymaa S, Kaivo-Oja N, Dragovic RA, et al (2006) Molecular basis of oocyte-paracrine signalling that promotes granulosa cell proliferation. J. Cell Sci. 119: 3811–3821. 10.1242/jcs.03105 16926195

[7] Knight PG, Glister C (2006) TGF-beta superfamily members and ovarian follicle development. Reproduction 132: 191–206. 16885529 10.1530/rep.1.01074

[8] Paulini F, Melo EO (2011) The role of oocyte-secreted factors GDF9 and BMP15 in follicular development and oogenesis. Reprod Domest Anim. 46: 354–361. 10.1111/j.1439-0531.2010.01739.x 21198974

[9] Pangas SA, Jorgez CJ, Tran M, Agno J, Li X, et al (2007) Intraovarian activins are required for female fertility. Mol Endocrinol. 21: 2458–2471. 10.1210/me.2007-0146 17609433

[10] Pernasetti F, Vasilyev VV, Rosenberg SB, Bailey JS, Huang HJ, et al (2001) Cell-specific transcriptional regulation of follicle-stimulating hormone-beta by activin and gonadotropin-releasing hormone in the LbetaT2 pituitary gonadotrope cell model. Endocrinology 142: 2284–2295. 11356674 10.1210/endo.142.6.8185

[11] Suszko MI, Lo DJ, Suh H, Camper SA, Woodruff TK (2003) Regulation of the rat follicle-stimulating hormone beta-subunit promoter by activin. Mol Endocrinol. 17: 318–332. 10.1210/me.2002-0081 12554780

[12] Kipp JL, Golebiowski A, Rodriguez G, Demczuk M, Kilen SM, et al (2011) Gene expression profiling reveals Cyp26b1 to be an activin regulated gene involved in ovarian granulosa cell proliferation. Endocrinology 152: 303–312. 21084447 10.1210/en.2010-0749 PMC3033060

[13] McLaughlin M, Bromfield JJ, Albertini DF, Telfer EE (2010) Activin promotes follicular integrity and oogenesis in cultured pre-antral bovine follicles. Mol Hum Reprod. 16: 644–653. 10.1093/molehr/gaq021 PMC2930516 20203128

[14] Rabinovici J, Spencer SJ, Jaffe RB (1990) Recombinant human activin-A promotes proliferation of human luteinized preovulatory granulosa cells in vitro. J Clin Endocrinol Metab 71: 1396–1398. 2229297 10.1210/jcem-71-5-1396

[15] Abe Y, Minegishi T, Leung PC (2004) Activin receptor signaling. Growth Factors 22, 105–110. 10.1080/08977190410001704688 15253386

[16] Bagga S, Bracht J, Hunter S, Massirer K, Holtz J, et al (2005) Regulation by let-7 and lin-4 miRNAs results in target mRNA degradation. Cell 122: 553–563. 16122423 10.1016/j.cell.2005.07.031

[17] Pillai RS, Bhattacharyya SN, Artus CG, Zoller T, Cougot N, et al.. (2005) Inhibition of translational initiation by Let-7 MicroRNA in human cells. Science 309, 1573–1576. 10.1126/science.1115079 16081698

[18] Bernstein E, Kim SY, Carmell MA, Murchison EP, Alcorn H, et al (2003) Dicer is essential for mouse development. Nat Genet. 35: 215–217. 10.1038/ng1253 14528307

[19] Li Q, Bian S, Hong J, Kawase-Koga Y, Zhu E, et al (2011) Timing specific requirement of microRNA function is essential for embryonic and postnatal hippocampal development. PLoS One 6: e26000. 21991391 10.1371/journal.pone.0026000 PMC3186801

[20] Yang WJ, Yang DD, Na S, Sandusky GE, Zhang Q, et al (2005) Dicer is required for embryonic angiogenesis during mouse development. J Biol Chem. 280: 9330–9935. 10.1074/jbc.M413394200 15613470

[21] Hong X, Luense LJ, McGinnis LK, Nothnick WB, Christenson LK (2008) Dicer1 is essential for female fertility and normal development of the female reproductive system. Endocrinology 149: 6207–6212. 18703631 10.1210/en.2008-0294 PMC2613048

[22] Nagaraja AK, Andreu-Vieyra C, Franco HL, Ma L, Chen R, et al (2008) Deletion of Dicer in somatic cells of the female reproductive tract causes sterility. Mol Endocrinol. 22: 2336–2352. 10.1210/me.2008-0142 PMC2582529 18687735

[23] Otsuka M, Zheng M, Hayashi M, Lee JD, Yoshino O, et al (2008) Impaired microRNA processing causes corpus luteum insufficiency and infertility in mice. J Clin Invest. 118: 1944–1954. 10.1172/JCI33680 PMC2289794 18398510

[24] Carletti MZ, Christenson LK (2009) MicroRNA in the ovary and female reproductive tract. J Anim Sci. 87: E29–38. 10.2527/jas.2008-1331 PMC3118666 18791135

[25] Singh SK, Pal BM, Girschick HJ, Bhadra U (2008) MicroRNAs–micro in size but macro in function. FEBS J 275: 4929–4944. 18754771 10.1111/j.1742-4658.2008.06624.x

[26] Debernardi S, Skoulakis S, Molloy G, Chaplin T, Dixon-McIver A, et al (2007) MicroRNA miR-181a correlates with morphological sub-class of acute myeloid leukaemia and the expression of its target genes in global genome-wide analysis. Leukemia 21: 912–916. 17330104 10.1038/sj.leu.2404605

[27] Naguibneva I, Ameyar-Zazoua M, Polesskaya A, Ait-Si-Ali S, Groisman R, et al (2006) The microRNA miR-181 targets the homeobox protein Hox-A11 during mammalian myoblast differentiation. Nat Cell Biol. 8: 278–284. 10.1038/ncb1373 16489342

[28] Li QJ, Chau J, Ebert PJ, Sylvester G, Min H, et al (2007) miR-181a is an intrinsic modulator of T cell sensitivity and selection. Cell 129: 147–161. 17382377 10.1016/j.cell.2007.03.008

[29] Sirotkin AV, Laukova M, Ovcharenko D, Brenaut P, Mlyncek M (2010) Identification of microRNAs controlling human ovarian cell proliferation and apoptosis. J Cell Physiol. 223: 49–56. 10.1002/jcp.21999 20039279

[30] Wang Y, Yu Y, Tsuyada A, Ren X, Wu X, et al (2011) Transforming growth factor-beta regulates the sphere-initiating stem cell-like feature in breast cancer through miRNA-181 and ATM. Oncogene 30: 1470–1480. 21102523 10.1038/onc.2010.531 PMC3063856

[31] Itano N, Atsumi F, Sawai T, Yamada Y, Miyaishi O, et al (2002) Abnormal accumulation of hyaluronan matrix diminishes contact inhibition of cell growth and promotes cell migration. Proc Natl Acad Sci USA 99: 3609–3614. 11891291 10.1073/pnas.052026799 PMC122571

[32] Xu Y, Liu L, Qiu X, Jiang L, Huang B, et al (2011) CCL21/CCR7 promotes G2/M phase progression via the ERK pathway in human non-small cell lung cancer cells. PLoS One 6: e21119. 21698152 10.1371/journal.pone.0021119 PMC3116867

[33] Robker RL, Richards JS (1998) Hormone-induced proliferation and differentiation of granulosa cells: a coordinated balance of the cell cycle regulators cyclin D2 and p27Kip1. Mol Endocrinol. 12: 924–940. 10.1210/mend.12.7.0138 9658398

[34] Mukasa C, Nomura M, Tanaka T, Tanaka K, Nishi Y, et al (2003) Activin signaling through type IB activin receptor stimulates aromatase activity in the ovarian granulosa cell-like human granulosa (KGN) cells. Endocrinology 144: 1603–1611. 12639945 10.1210/en.2002-220978

[35] Park Y, Maizels ET, Feiger ZJ, Alam H, Peters CA, et al (2005) Induction of cyclin D2 in rat granulosa cells requires FSH-dependent relief from FOXO1 repression coupled with positive signals from Smad. J Biol Chem. 280: 9135–9148. 10.1074/jbc.M409486200 PMC1564190 15613482

[36] Kipp JL, Kilen SM, Woodruff TK, Mayo KE (2007) Activin regulates estrogen receptor gene expression in the mouse ovary. J Biol Chem. 282: 36755–36765. 10.1074/jbc.M705143200 17951260

[37] Bennett J, Wu YG, Gossen J, Zhou P, Stocco C (2012) Loss of GATA-6 and GATA-4 in Granulosa Cells Blocks Folliculogenesis, Ovulation, and Follicle Stimulating Hormone Receptor Expression Leading to Female Infertility. Endocrinology 153: 2474–2485. 22434075 10.1210/en.2011-1969 PMC3339651

[38] Kidder GM, Vanderhyden BC (2010) Bidirectional communication between oocytes and follicle cells: ensuring oocyte developmental competence. Can J Physiol Pharmacol 88: 399–413. 20555408 10.1139/y10-009 PMC3025001

[39] Pelusi C, Ikeda Y, Zubair M, Parker KL (2008) Impaired follicle development and infertility in female mice lacking steroidogenic factor 1 in ovarian granulosa cells. Biol Reprod. 79: 1074–1083. 10.1095/biolreprod.108.069435 PMC2780474 18703422

[40] Fiedler SD, Carletti MZ, Hong X, Christenson LK (2008) Hormonal regulation of MicroRNA expression in periovulatory mouse mural granulosa cells. Biol Reprod 79: 1030–1037. 18716288 10.1095/biolreprod.108.069690 PMC2780477

[41] Carletti MZ, Fiedler SD, Christenson LK (2010) MicroRNA 21 blocks apoptosis in mouse periovulatory granulosa cells. Biol Reprod 83: 286–295. 20357270 10.1095/biolreprod.109.081448 PMC2907287

[42] Lin F, Li R, Pan ZX, Zhou B, Yu DB, et al (2012) miR-26b promotes granulosa cell apoptosis by targeting ATM during follicular atresia in porcine ovary. PLoS One 7: e38640. 22737216 10.1371/journal.pone.0038640 PMC3380909

[43] Yang X, Zhou Y, Peng S, Wu L, Lin HY, et al (2012) Differentially expressed plasma microRNAs in premature ovarian failure patients and the potential regulatory function of mir-23a in granulosa cell apoptosis. Reproduction 144: 235–244. 22653319 10.1530/REP-11-0371

[44] Lee SI, Lee BR, Hwang YS, Lee HC, Rengaraj D, et al (2011) MicroRNA-mediated posttranscriptional regulation is required for maintaining undifferentiated properties of blastoderm and primordial germ cells in chickens. Proc Natl Acad Sci U S A 108: 10426–10431. 21670268 10.1073/pnas.1106141108 PMC3127938

[45] Lingenfelter BM, Tripurani SK, Tejomurtula J, Smith GW, Yao J (2011) Molecular cloning and expression of bovine nucleoplasmin 2 (NPM2): a maternal effect gene regulated by miR-181a. Reprod Biol Endocrinol 9: 40. 21447182 10.1186/1477-7827-9-40 PMC3072940

[46] Matzuk MM, Kumar TR, Bradley A (1995) Different phenotypes for mice deficient in either activins or activin receptor type II. Nature 374: 356–360. 7885474 10.1038/374356a0

[47] Dragovic RA, Ritter LJ, Schulz SJ, Amato F, Thompson JG, et al (2007) Oocyte-secreted factor activation of SMAD 2/3 signaling enables initiation of mouse cumulus cell expansion. Biol Reprod. 76: 848–857. 10.1095/biolreprod.106.057471 17192514

[48] Pedersen T (1969) Follicle growth in the immature mouse ovary. Acta Endocrinol (Copenh) 62: 117–132. 5394283 10.1530/acta.0.0620117

[49] Shelling AN (2010) Premature ovarian failure. Reproduction 140: 633–641. 20716613 10.1530/REP-09-0567

[50] Nishi Y, Yanase T, Mu Y, Oba K, Ichino I, et al (2001) Establishment and characterization of a steroidogenic human granulosa-like tumor cell line, KGN, that expresses functional follicle-stimulating hormone receptor. Endocrinology 142: 437–445. 11145608 10.1210/endo.142.1.7862

[51] Kipp JL, Kilen SM, Bristol-Gould S, Woodruff TK, Mayo KE (2007) Neonatal exposure to estrogens suppresses activin expression and signaling in the mouse ovary. Endocrinology 148: 1968–1976. 17255206 10.1210/en.2006-1083

[52] Wang S, Aurora AB, Johnson BA, Qi X, McAnally J, et al. The endothelial-specific microRNA miR-126 governs vascular integrity and angiogenesis. Dev Cell. 15(2): 261–271. 10.1016/j.devcel.2008.07.002 PMC2685763 18694565

[53] Li R, Yan G, Li Q, Sun H, Hu Y, et al (2012) MicroRNA-145 Protects Cardiomyocytes against Hydrogen Peroxide (H(2)O(2))-Induced Apoptosis through Targeting the Mitochondria Apoptotic Pathway. PLoS One 7: e44907. 23028672 10.1371/journal.pone.0044907 PMC3445575

[54] Jiang Y, Hu Y, Zhao J, Zhen X, Yan G, et al (2011) The orphan nuclear receptor Nur77 regulates decidual prolactin expression in human endometrial stromal cells. Biochem Biophys Res Commun 404: 628–633. 21146499 10.1016/j.bbrc.2010.12.027

[55] Tang F, Hajkova P, Barton SC, Lao K, Surani MA (2006) MicroRNA expression profiling of single whole embryonic stem cells. Nucleic Acids Res. 34: e9. 10.1093/nar/gnj009 PMC1351374 16434699

[56] Li YL, Ye F, Hu Y, Lu WG, Xie X (2009) Identification of suitable reference genes for gene expression studies of human serous ovarian cancer by real-time polymerase chain reaction. Anal Biochem 394: 110–116. 19622337 10.1016/j.ab.2009.07.022

[57] O’Connor T, Wilmut I, Taylor J (2012) Quantitative Evaluation of Reference Genes for Real-Time PCR During In Vitro Maturation of Ovine Oocytes. Reprod Domest Anim. 10.1111/rda.12112 23066791

[58] Livak KJ, Schmittgen TD (2001) Analysis of relative gene expression data using real-time quantitative PCR and the 2(-Delta Delta C(T)) Method. Methods 25: 402–408. 11846609 10.1006/meth.2001.1262

[59] Sun H, Chen L, Yan G, Wang R, Diao Z, et al (2009) HOXA10 suppresses p/CAF promoter activity via three consecutive TTAT units in human endometrial stromal cells. Biochem Biophys Res Commun 379: 16–21. 19084499 10.1016/j.bbrc.2008.11.144

