# Supplementary figures and images for: MicroRNA-181a Suppresses Mouse Granulosa Cell Proliferation by Targeting Activin Receptor IIA
Source: PLoS One. 2013 Mar 20;8(3):e59667. doi: 10.1371/journal.pone.0059667 (PMC3604175; doi:10.1371/journal.pone.0059667)

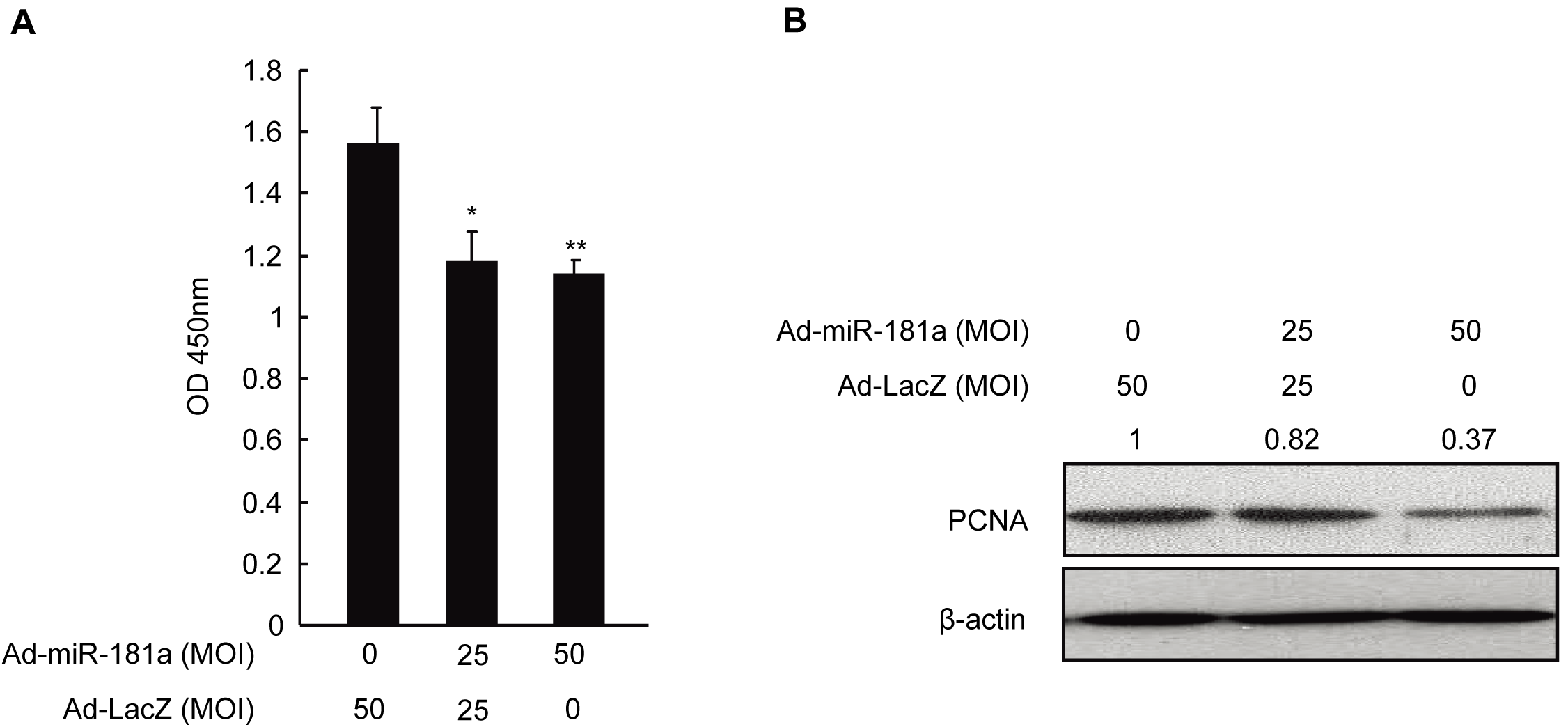

Supplement: Figure S1 — Effect of miR-181a on KGN cell proliferation. KGN cells were infected with Ad-miR-181a (MOI = 0, 25, and 50) for 48 h. (A) The proliferation of KGN cells was measured by CCK-8. (B) Protein level of PCNA was examined by Western blotting. Relative protein levels were measured by densitometry using Quantity One Software and normalized to β-actin, Ad-LacZ group; the ratios were presented above the Western blot bands. *p<0.05, **p<0.01, compared with control groups. (TIF) [file pone.0059667.s001.tif]

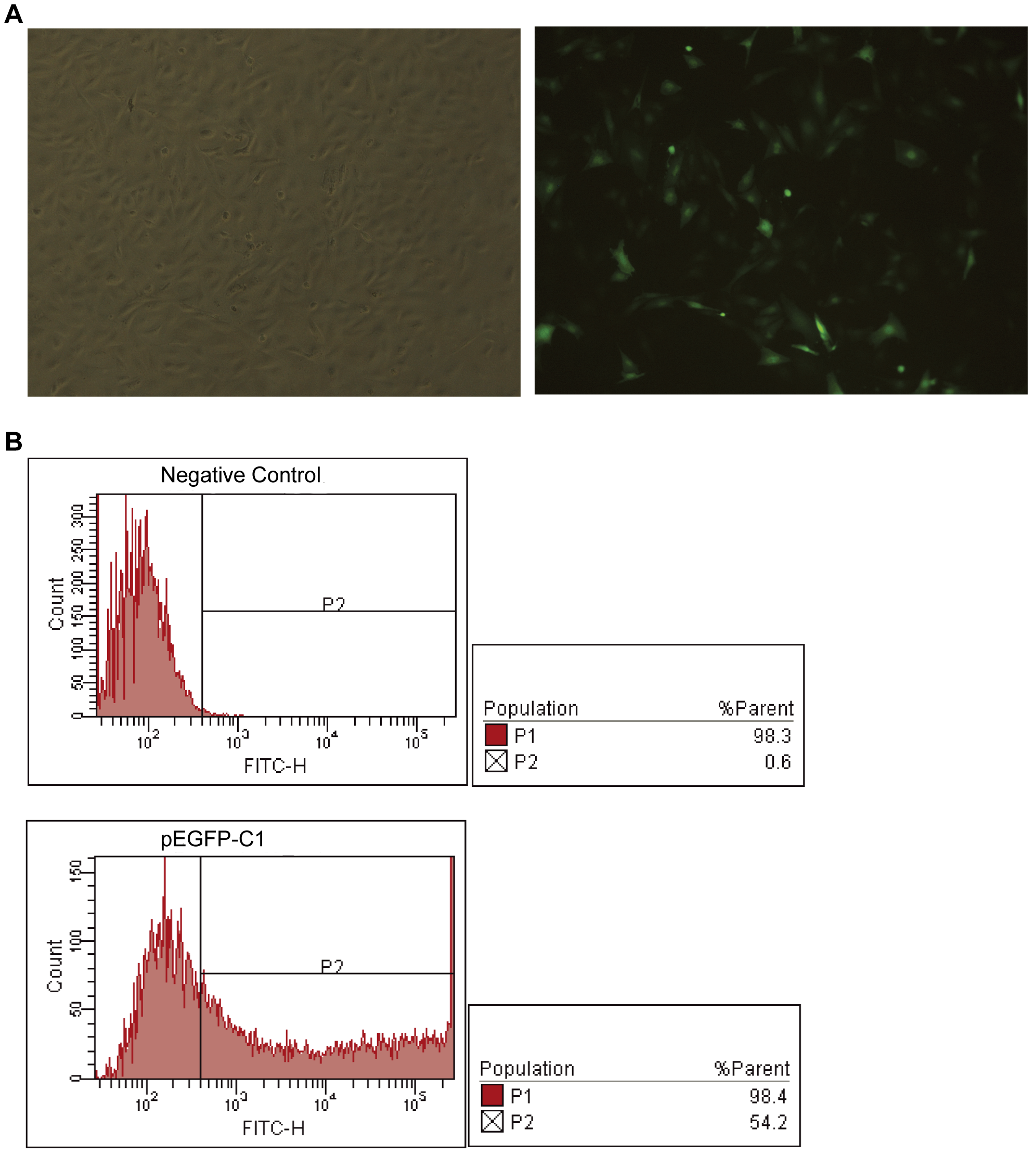

Supplement: Figure S2 — Transfection efficiencies of mGC detected by FCM after transfection with pEGFP-C1 plasmid for 48 h. mGC in 60-mm dish was transfected with 3 µg pEGFP-C1 plasmid. After 48 h, GFP fluorescence (A) and flow cytometry (B) were performed to measure the transfection efficiencies of mGC. (TIF) [file pone.0059667.s002.tif]

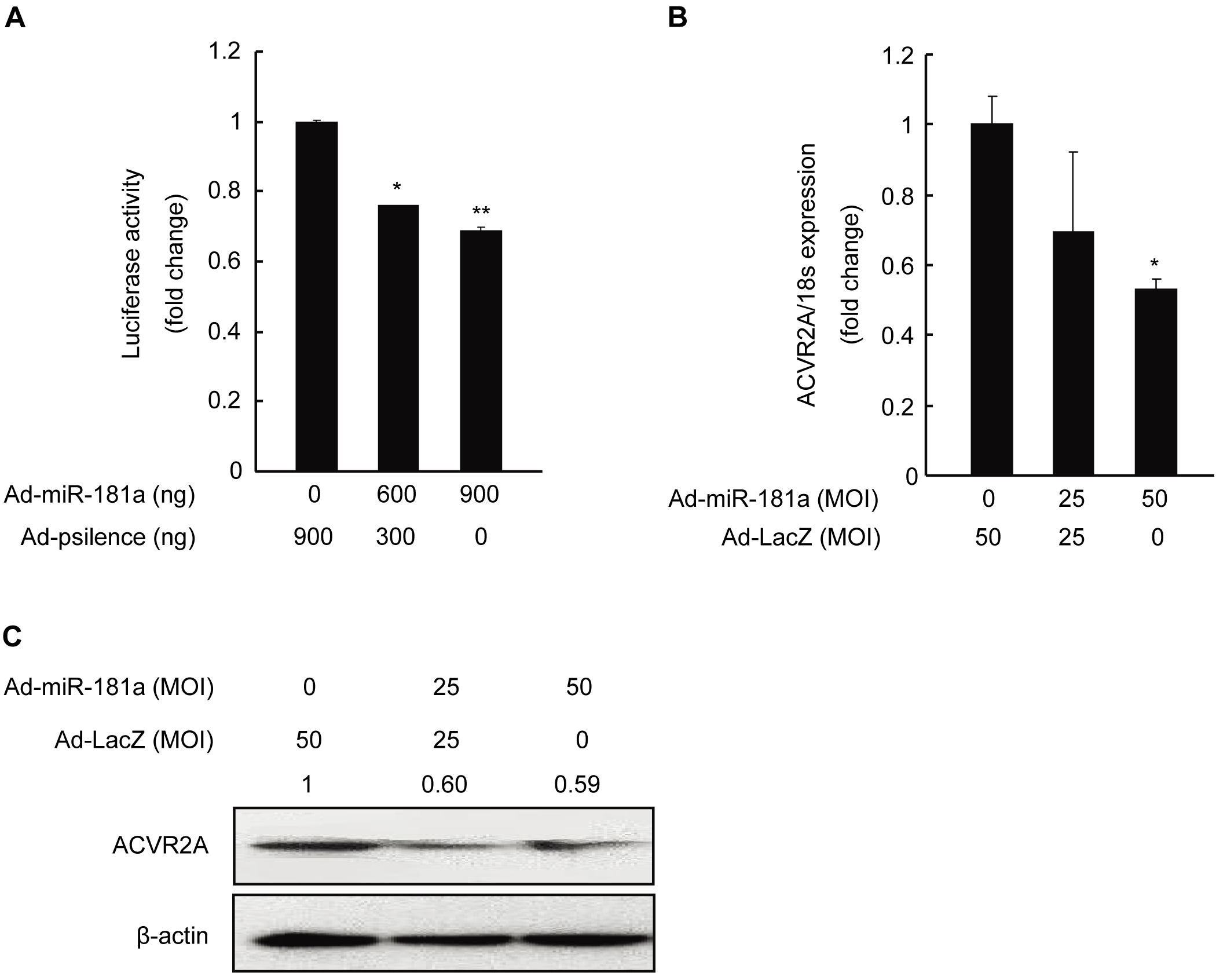

Supplement: Figure S3 — Identification of ACVR2A as a target gene of miR-181a in KGN cells. (A) The 3′-UTR luciferase activity of the human ACVR2A gene was examined in HEK293T cells after overexpression of miR-181a. ACVR2A mRNA (B) and protein (C) levels were measured by qRT-PCR and Western blotting in KGN cells infected with Ad-miR-181a for 48 h. Relative protein levels were measured by densitometry using Quantity One Software and normalized to β-actin, Ad-LacZ group; the ratios were presented above the Western blot bands. *p<0.05, **p<0.01, compared with controls. (TIF) [file pone.0059667.s003.tif]

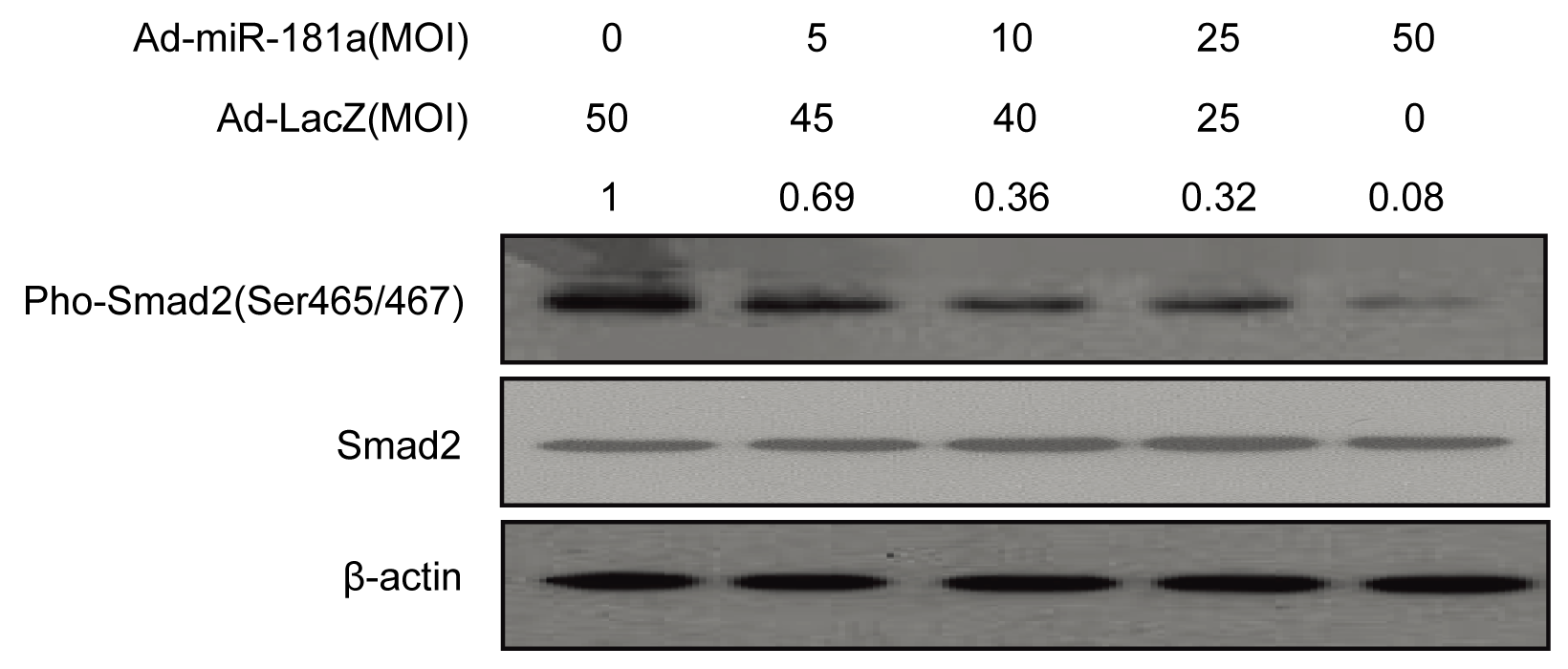

Supplement: Figure S4 — Inhibition of the phosphorylation of Smad2 in KGN cells by miR-181a. Western blot analysis of the levels of Smad2 and phosphorylated Smad2 (Ser465/467) in KGN cells treated with Ad-miR-181a for 24 h. Relative protein levels of phosphorylated Smad2 were measured by densitometry using Quantity One Software and normalized to β-actin, Ad-LacZ group; the ratios were presented above the Western blot bands. (TIF) [file pone.0059667.s004.tif]

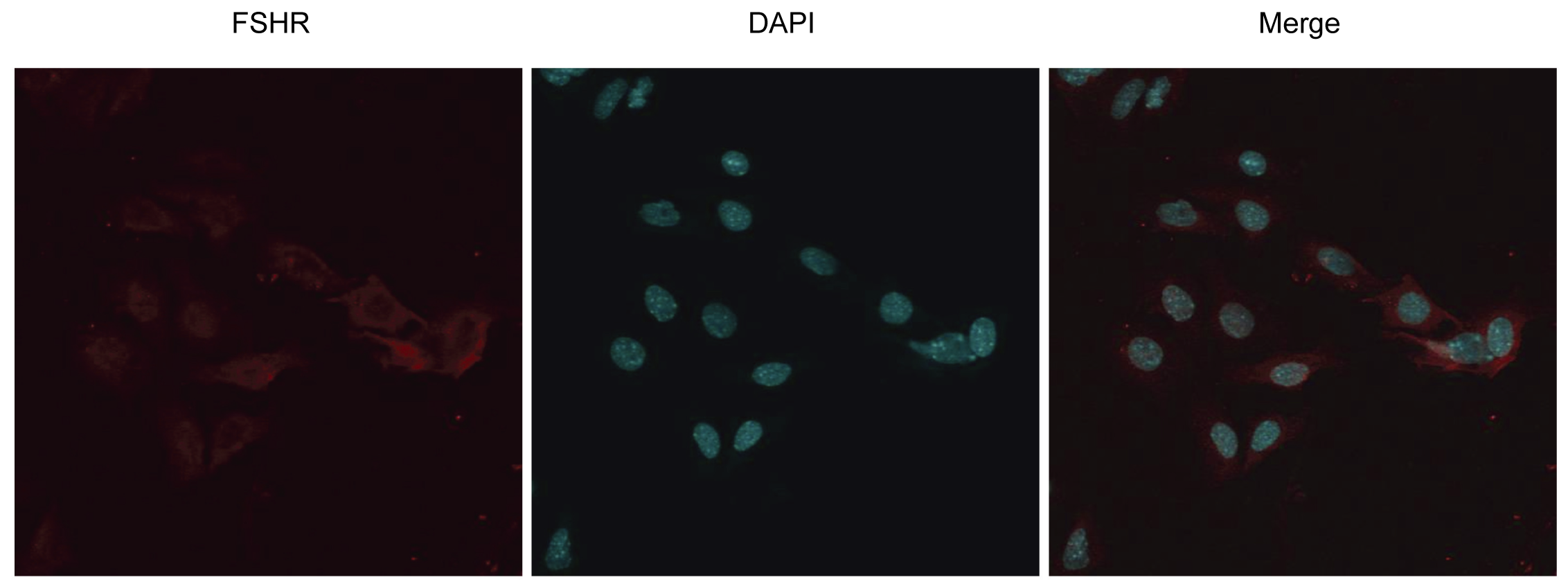

Supplement: Figure S5 — Measurement of follicle stimulating hormone receptor (FSHR) by immunofluorescent staining. Primary granulosa cells were isolated from 21-day-old mouse ovaries. FSHR protein was measured by immunofluorescent staining (FSHR: red; DAPI: blue). (TIF) [file pone.0059667.s005.tif]
