# Supplementary material for: MicroRNA-181a Suppresses Mouse Granulosa Cell Proliferation by Targeting Activin Receptor IIA
Source: PLoS One. 2013 Mar 20;8(3):e59667. doi: 10.1371/journal.pone.0059667 (PMC3604175; doi:10.1371/journal.pone.0059667)
Supplement: Table S1 — FSH levels in premature ovarian failure (POF) patients and normal females. (DOC) [file pone.0059667.s006.doc]

**Supporting Information tables**

Table S1. FSH levels in premature ovarian failure (POF) patients and normal females.

|  | Normal (n = 11) | POF (n = 8) |
| --- | --- | --- |
| Age (years) | 30.27 ± 3.77 | 30 ± 2.60 |
| FSH (IU/L) | 7.01 ± 3.56 | 80.30 ± 19.56 ** |

**p<0.01, compared with the control.
