# Supplementary material for: MicroRNA-181a Suppresses Mouse Granulosa Cell Proliferation by Targeting Activin Receptor IIA
Source: PLoS One. 2013 Mar 20;8(3):e59667. doi: 10.1371/journal.pone.0059667 (PMC3604175; doi:10.1371/journal.pone.0059667)
Supplement: Table S2 — Oligonucleotide primer sequences of quantitative real-time PCR. (DOC) [file pone.0059667.s007.doc]

Table S2. Oligonucleotide primer sequences of quantitative real-time PCR.

| **Gene** | **Forward primer (5’🡪3’)** | **Reverse primer (5’🡪3’)** |
| --- | --- | --- |
| **Mouse gene** |  |  |
| Cyclin D2 | ACACCGACAACTCTGTGAAGC | GCCAGGTTCCACTTCAGCTTA |
| Acvr2a | ACACAGCCCACTTCAAATCC | AACCAAATCTTCCCCTTGCT |
| CYP19A1 | GACACATCATGCTGGACACC | CAAGTCCTTGACGGATCGTT |
| P450scc | CAGACGCATCAAGCAGCAA | CTGGAGGCAGGTTGAGCAT |
| ESR1 | AAGGGCAGTCACAATGAACC | GCCAGGTCATTCTCCACATT |
| 18s | ATGGCCGTTCTTAGTTGGTG | CGGACATCTAAGGGCATCAC |
| **Human gene** |  |  |
| ACVR2A | AAAAGATGGCCACAAACCTG | CCAACCTGTCCATGGGTATC |
| 18s | CGGCTACCACATCCAAGGAA | CTGGAATTACCGCGGCT |
